# Microvascular arteriolar reactivity in response to skeletal muscle contractions in female and male hamsters

**DOI:** 10.14814/phy2.70655

**Published:** 2025-11-28

**Authors:** Nicole M. Fletcher, Coral L. Murrant

**Affiliations:** ^1^ Department of Human Health and Nutritional Sciences University of Guelph Guelph Ontario Canada

**Keywords:** active hyperaemia, arteriole, muscle contraction, skeletal muscle, vasodilation

## Abstract

Microvascular control mechanisms involved in the blood flow response to muscle contractions have been well documented in males but remain poorly understood in females. Therefore, we characterized arteriolar vasodilation in situ using intravital microscopy of the retractor muscle of anesthetized female and male hamsters (8–13 weeks). Arterioles were stimulated physiologically by contracting 3–5 skeletal muscle fibers overlying the arteriole for 2 min using a range of twitch and tetanic stimulation parameters: 6, 15, and 60 contractions per minute (cpm) at 20 Hz, or 4, 20, and 70 Hz at 15 cpm (250 ms train duration) and pharmacologically via 2 min micropipette application of nitric oxide (NO, 10^−5^ M), adenosine (ADO, 10^−6^ M) and potassium (K^+^, 20 mM) as well as acetylcholine (ACh, 10^−6^ M) to assess local and conducted responses. Sex differences were not observed in the magnitude or rate of arteriolar vasodilation under any physiological or pharmacological condition. Collectively, these data demonstrate that arteriolar reactivity to muscle contractions and to pharmacological stimuli relevant to muscle contractions, were not different between females and males. These data suggest that the integrated vascular response during active hyperemia may not be sexually dimorphic.

## 1 INTRODUCTION

During exercise, skeletal muscle oxygen consumption can increase 10‐ to 15‐ fold (Joyner & Casey, 2015). This increase in metabolic rate requires a compensatory increase in blood flow to match oxygen delivery to substrate demand to maintain proper cellular function, and prevent fatigue and cellular damage. Blood flow in skeletal muscle has been shown to be linearly related to a wide range of metabolic demands (i.e., Bockman, 1983; Hamann et al., 2004; Radegran & Saltin, 1998). The integration of both central processes (i.e., baro‐ and pressor receptor responses, changes in cardiac output, sympathetic redistribution of blood flow, etc.) and mechanisms local to the tissue (i.e., local vasodilator production, myogenic response, flow‐induced vasodilation) are required to facilitate blood flow adjustments in response to muscle contraction. Interestingly, the linear relationship between blood flow and metabolic demand has been demonstrated in the absence of central neural influence (Bockman, 1983; Heinonen et al., 2013) emphasizing the importance of the tissue‐level mechanisms to the overall active hyperaemic response.

The local processes that establish the active hyperaemic response include processes whereby contracting skeletal muscle fibers communicate with the vascular network, and blood vessel intercommunication to direct blood flow to the capillaries supplying active skeletal muscle fibers. These processes include the release of vasodilatory products generated by contracting skeletal muscle and changes in metabolism (i.e., nitric oxide [NO], purines [ATP], and adenosine [ADO] and potassium [K^+^]) and products released from associated cells such as endothelial cells (i.e., prostaglandins, NO, and EDHF) and red blood cells (i.e., ATP), which then diffuse to vascular endothelial cells or vascular smooth muscle cells to cause vasodilation (for review see (Joyner & Casey, 2015; Sarelius & Pohl, 2010)). Stimulation of the cells of the vasculature also initiates longitudinal intervascular communication (conducted responses) along the vascular wall to coordinate the network vasodilatory response (Bagher & Segal, 2011) and increase perfusion of capillaries supplying the active muscle fibers. Other local processes involved in active hyperaemia include flow‐mediated responses, the myogenic response and sympatholysis (for review see (Sarelius & Pohl, 2010)). Understanding the contribution of each of these processes to the active hyperaemic response and how they integrate to match blood flow to metabolic demand is complex and complicated by the idea that redundancy between processes occurs, whereby one process can take the place of another process (Lamb & Murrant, 2015). Further, our current understanding of the complexity of active hyperaemia has been through research done predominantly in male models, and the understanding of these processes in females is in its infancy.

Sexual dimorphisms in local microvascular responses related to active hyperaemia and blood flow control have been demonstrated but the results are not yet definitive. For example, evidence for sex differences have been observed in the variable contributions of NO, prostaglandins and EDHF to flow‐mediated responses in skeletal muscle arterioles (Wu et al., 2001) and wall shear stress in rat tail artery (Pak et al., 2002). Sex differences in the myogenic response have been demonstrated in cerebral, coronary and skeletal muscle (Geary et al., 1998; Huang et al., 1997; Jeffrey et al., 2025; Wellman et al., 1996) although not consistently (Horn, Morrison, et al., 2025; Skarsgard et al., 1997). Further, differences in vascular reactivity between males and females have been identified with acetylcholine (ACh) in some (Dias et al., 2014; Mayhan et al., 2002; Sanchez et al., 1996) but not all studies (Horn, White, et al., 2025; Shechtman & Katovich, 1993). The vascular response to KCl has been shown to be sexually dimorphic (Dias et al., 2014; McCulloch & Randall, 1998; Sanchez et al., 1996; Shechtman & Katovich, 1993) but not unilaterally (Horn, Morrison, et al., 2025). And a lack of sex differences in the vascular response to NO donors has been demonstrated (Bell et al., 1995; Dias et al., 2014; Horn, White, et al., 2025; McCulloch & Randall, 1998; Skarsgard et al., 1997) but not in all cases (Mayhan et al., 2002). Sex differences in vascular reactivity have been shown to be partially attributable to the enhanced release and/or activity of endothelial‐dependent NO related to the presence of estrogen in females (Bell et al., 1995; Geary et al., 2000; Skarsgard et al., 1997; Wellman et al., 1996) but also may be due to fundamental sex differences in cell signaling mechanisms responsible for generating the vascular responses (Dias et al., 2014; Jeffrey et al., 2025). Therefore, given the potential for sex differences in the vascular responses and processes related to active hyperaemia, it is highly likely that the arteriolar microvascular response to muscle contraction will be sexually dimorphic, but whether the microvascular arteriolar vascular response to muscle contractions differs between males and females is not known.

Additionally, evidence suggests that there may be sex differences in skeletal muscle contraction and metabolic processes, which may be the source of vasodilators that communicate with the microvasculature during active hyperemia. Specific contractile force generated at the tissue level has been shown to differ between females and males in some (Bisschop et al., 1997; Lafoux et al., 2020), but not all studies (Daniels et al., 2008; Hill et al., 2020; Khurram et al., 2018; Teigen et al., 2020) and sex differences in mitochondrial oxidative bioenergetics (Cardinale et al., 2018; Miotto et al., 2018) and resting oxidative metabolism (Ansdell et al., 2019; Citherlet et al., 2024; Fellahi et al., 2014; Keller et al., 2023; Landers‐Ramos et al., 2024; Molbo et al., 2022; Rasica et al., 2022; Zaleski et al., 2023) have been identified. Therefore, if contraction and metabolism are sources of metabolic vasodilatory byproducts that signal the vasculature during active hyperemia, then the communicating molecules used to signal the microvasculature during contraction may differ between males and females. Currently, whether sexual dimorphisms in contractile function could translate into differing vascular responses between males and females during muscle contraction is unknown.

Therefore, the purpose of the current investigation was to determine whether the microvascular arteriolar response to muscle contractions were sexually dimorphic. We used intravital microscopy of male and female hamster retractor muscle in situ to observe the change in diameter of arterioles overlying contracting skeletal muscle fibers stimulated to contract over a wide range of contraction parameters. Further, to better understand potential sex differences in vascular reactivity we directly exposed male and female microvascular arterioles to vasodilators associated with contraction‐induced vasodilation (ADO, K^+^, and NO). We hypothesized that the arteriolar response to muscle contractions would be sexually dimorphic as would the response to pharmacologically applied agonists. Finally, for a clearer interpretation of our vascular response data in the face of potential influences of sex differences in muscle contraction we compared contractile force and fatigue of retractor muscle in vitro.

## 2 METHODS

### 2.1 Ethics statement and animals

All procedures were approved by the Animal Care Committee at the University of Guelph and were performed in accordance with the Canadian Council of Animal Care guidelines. All animals were provided with continuous access to food (Envigo‐Harlan Teklad Food, Brampton, ON, Canada; diet 2914, T2914.15) and water. Following each experiment, animals were euthanized with an overdose of sodium pentobarbital (0.26 mg/mL via an intravenous femoral catheter) to effect. Adult female (90–142 g) and male (93–143 g) Golden Syrian hamsters (Envigo, Michigan, USA) aged 8–13 weeks were used for all experimental protocols. Golden Syrian hamsters differ from other common rodents, such as rats and mice, in that they are fossorial. This unique behavior is associated with physiological adaptations in the oxygen transport pathway, including distinct ventilatory responses under hypoxia, and hypercapnia (Frappell & Mortola, 1994; Walker et al., 1985), and higher baseline hematocrit levels compared to rats at lower altitudes (Walker et al., 1982), which may reflect adaptations to low‐oxygen burrow environments. Additionally, sexual dimorphisms in body weight are minimal in early adulthood but manifest with age, with females generally larger than males. In the current study we used adult animals where a sexual dimorphism in size had not yet been expressed.

### 2.2 Retractor exteriorization for in situ experiments

Animals were anesthetized with sodium pentobarbital (70 mg/kg) via an intraperitoneal injection and tracheotomized. A polyethylene catheter (outer tip diameter approximately 0.5 mm) was placed in the left femoral artery for supplemental sodium pentobarbital infusion (10 mg/mL saline, 0.56 mL/h) during experimental protocols. To confirm that animals remained within the surgical plane of anesthesia throughout the experiment, respiratory rate and depth were monitored continuously and the absence of withdrawal from a toe pinch was verified at regular intervals. The animal was transferred onto an acrylic platform and esophageal temperature was maintained at 37°C via convective heat from a coiled water‐filled glass tube (42°C) secured underneath the animal.

**FIGURE 1:**
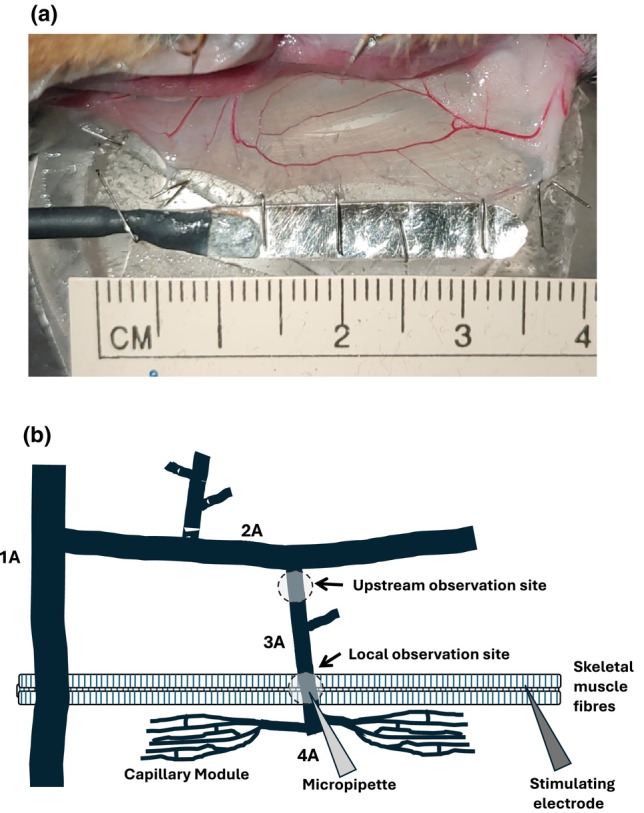
Exteriorized retractor muscle and microvascular arteriolar network experimental site. (a) An image of the exteriorized retractor preparation with the vascular network intact. The retractor muscle is pinned on a lucite plate with a ground electrode secured longitudinally along the base of the muscle for in situ muscle contraction experiments. (b) Schematic of part of the retractor arteriolar microvascular network experimental site depicting a feed arteriole (1A), and branching 2A, 3A, and 4A arterioles prior to capillary modules. The diagram indicates the experimental set‐up of different protocols including the local 3A arteriolar site for micropipette application and local observation site during ACh, ADO, NO, and K^+^ application as well as the upstream observation site during ACh application. Also depicted are a bundle of muscle fibers underlaying the 3A arteriolar observation site that were stimulated for protocols investigating the arteriolar response (local observation site) to muscle contractions. Schematic is not drawn to scale.

The right retractor muscle was prepared for intravital microscopy as originally described (Priddy & Brodie, 1948; Sullivan & Pittman, 1982) and modified to preserve the vascular connections to the muscle to ensure perfusion of the entire muscle once exteriorized. Briefly, with the animal in the prone position a ~6 cm incision was made through the skin to expose the retractor muscle. The dorsal surface of the retractor was cleared of connective tissue and adipose tissue briefly, with care not to touch the muscle. The animal was rotated on its right side, the right retractor was gently reflected laterally to expose the ventral surface of the muscle. The muscle was spread over a semicircular lucite plate and pinned into place using insect pins (Figure 1a). Careful consideration was taken during this process to ensure that the muscle was not overstretched and that blood flow in the main feed arteries and veins were not altered by physical distortion of the preparation. Average sarcomere length of exteriorized muscles were 2.5 ± 0.33 μm. The ventral surface of the retractor muscle, with its origin and insertion still intact, was carefully freed of connective tissue and adipose tissue without touching the surface of the muscle. The exposed areas of the animal's back and edges of the reflected muscle were covered in lint‐free Kimwipes to keep these parts of the exposed tissue wet and prevent drying. During the exteriorization process and experiments, the muscle was continuously superfused with a standard bicarbonate‐buffered salt solution containing (in mM/L) 131.9 NaCl, 4.7 KCl, 2.0 CaCl_2_, 1.2 MgSO_4_, and 30 NaHCO_3_, and equilibrated with gas containing 5% CO_2_‐95% N_2_ to achieve a pH of solution between 7.35 and 7.45 and an osmolarity between 280 and 300 mOsm/L. Tubocurarine hydrochloride pentahydrate (0.3 mg/L, nicotinic cholinergic membrane receptor blocker; Sigma‐Aldrich, Oakville, ON, Canada) was added to the superfusate for all experiments. Tubocurarine (curare) was added to block nicotinic cholinergic membrane receptors on skeletal muscle cells to ensure that electrode stimulation of the muscle evoked contractions via directly depolarizing muscle fibers and not by stimulation of motor nerves within the tissue. Retractor muscle temperature was maintained at 34.0°C–34.5°C by heating the superfusate solution to 42°C and adjusting the drip rate to achieve the desired temperature. Following the retractor exteriorization, the hamster was transferred onto the microscope stage and allowed to equilibrate for 45–60 min prior to data collection.

### 2.3 Visualization of the microvascular network in situ using intravital microscopy

The microvascular network of the retractor preparation was visualized in situ via transillumination with a tungsten lamp and BX51WI microscope (Olympus Canada Inc., Richmond Hill, ON, Canada) using 10× (numerical aperture 0.30) and 20× (numerical aperture 0.40) long working distance objectives. A 10× objective was used when characterizing the arteriolar network and measuring distances between local stimulation sites and upstream observation sites of interest, which resulted in a final magnification of 540×. A 20× objective and 1.6 magnification changer were used to assess baseline and maximum diameters, and vascular reactivity to pharmacological and physiological stimuli. The final magnification of the site was approximately 2000×. The microscope image was displayed on a video monitor using a video camera (DC220; Dage‐MTI Inc., Michigan, MI, USA) and digitized using StreamCatcher Pro video compression software (StarTech, London, ON, Canada).

### 2.4 In situ vascular reactivity experiments

#### 2.4.1 Resting baseline diameters and maximum diameters across the arteriolar network

Baseline diameters and maximum diameters were assessed across the arteriolar network (2A, 3A, and 4A arterioles, Figure 1b). Resting baseline arteriolar diameters were recorded for 1 min in 2A, 3A, and 4A arterioles. Maximum diameters were quantified in the same vessels after 2 min of exposure to sodium nitroprusside (SNP, NO donor; Sigma‐Aldrich; Oakville, ON, Canada) at the end of each experiment (see below).

#### 2.4.2 Arteriolar reactivity in response to muscle contractions

Three to five skeletal muscle fibers were stimulated to contract using a silver wire microelectrode placed close to, but not touching, muscle fibers running perpendicular to a 3A arteriole (Figure 1b). The microelectrode was placed approximately 1000 μm away from the 3A arteriole of interest. A ground electrode was secured in place using bent insect pins around the longitudinal edge of the muscle and was exposed to the superfusate solution (Figure 1a). Each stimulus was a square wave pulse of 0.4 ms and 8–15 V (Grass S48 stimulator, Quincy, MA, USA). Voltage was determined daily at the beginning of each experiment and was adjusted until the muscle fibers appeared to be contracting maximally. Once voltage was determined it was kept constant throughout the duration of the experiment.

For each contraction protocol below, train duration was held constant at 250 ms while either stimulus frequency (number of impulses per train) was varied at a constant contraction frequency (15 contractions per minute [cpm]), or contraction frequency was varied at a constant stimulus frequency (20 Hz). In contraction protocol A: stimulus frequencies at 4, 20, and 70 Hz at a constant contraction frequency of 15 cpm were used to generate twitch contractions, submaximal (unfused tetanus) and maximal tetanic contractions (fused tetanus) as determined by the in vitro experiments using retractor muscle. In contraction protocol B: contraction frequencies at 6, 15, and 60 cpm at a constant stimulus frequency of 20 Hz (50% of maximal force production, determined for retractor muscle via in vitro experiment). These contraction frequencies represent low, intermediate, and high contraction frequencies respectively. 3A arteriolar diameters were continuously assessed for 1 min prior to muscle contractions, during the 2 min contraction period, and for 2 min of recovery following cessation of muscle contractions. A minimum of 5 min was allotted between each contraction condition to allow diameter to recover to resting baseline levels. Stimulus and contraction frequencies were randomized per protocol and per day. Only one 3A arteriole was stimulated via muscle contractions per experiment and both contraction protocols (A and B) were performed on each arteriole.

#### 2.4.3 Arteriolar reactivity in response to NO, ADO, and K^+^

We locally micropipette applied NO (10^−5^ M), ADO (10^−6^ M) or K^+^(20 mM) (agonists from Sigma‐Aldrich, Oakville, ON, Canada) to a 3A arteriole via micropipette application. Briefly, the vasoactive agonist was loaded into glass‐pulled micropipettes with a tip beveled to 8–12 μm and was placed as close to a 3A arteriole as possible without touching the muscle preparation using a micromanipulator (Narishige, East Meadow, NY, USA). A water manometer system pressurized at 30 cmH_2_O was used to eject the contents of the micropipette locally to a small region of the blood vessel (~200 μm) as previously described (Murrant & Sarelius, 2002; Twynstra et al., 2012). Fluorescein isothiocyanate‐dextran (100 μM, FITC; 4 kDa; Sigma‐Aldrich, Oakville, ON, Canada) was added to the micropipette solution so brief epifluorescence could be used to verify flow from the micropipette tip and to ensure the micropipette flowed over the local site and away from the upstream 3A observation site. Micropipette application of FITC alone did not affect 3A arteriolar diameter in females or males (data not shown). We applied concentrations of vasodilators that did not induce maximal vasodilation in the 3A arterioles tested. 3A arteriolar diameters were continuously assessed for 1 min prior to agonist exposure, during the 2 min exposure period, and for 2 min of recovery during wash‐out of the agonist.

#### 2.4.4 Local and conducted responses induced by ACh

Local arteriolar responses to ACh and upstream conducted responses were examined at the 3A arteriole in males and females. 3A arterioles were locally stimulated with 10^−6^ M ACh (Sigma‐Aldrich, Oakville, ON, Canada) via micropipette application using the same methodology described above.

Changes in arteriolar diameter were assessed at the local 3A arteriolar stimulation site as well as a 3A arteriole site approximately 1000 μm upstream from the local application site (Figure 1b). At local and upstream observation sites resting baseline diameter was recorded for 1 min prior to ACh exposure. The local 3A arteriole was then exposed to the contents of the micropipette for 2 min, followed by 2 min of recovery with no agonist exposure. A minimum of 5 min was allotted between each ACh application.

#### 2.4.5 Maximal diameters at the end of each in situ experiment

At the end of each experiment, maximal arteriolar diameters were recorded for 2 min at vascular observation sites of interest after exposing the entire preparation to 10^−3^ M SNP via the superfusate. This concentration of SNP is considered to produce maximal vasodilation in males (Murrant et al., 2014; Twynstra et al., 2012). However, in females it is not known if this concentration of SNP also elicits maximal vasodilation. Therefore, in a sub‐set of experiments in females (*n* = 9) maximal diameter was also recorded after 2 min exposure to a calcium‐free superfusate solution (with 10^−3^ M EGTA, ethylene glycol tetraacetic acid; Sigma‐Aldrich, Oakville, ON, Canada), which is known to elicit maximal vasodilation (Bolz et al., 2003). In these experiments the muscle preparation was superfused with either zero‐calcium superfusate solution or SNP, in a randomized order, followed by 2 min exposure to the second agonist, with maximum diameter recorded after each 2 min exposure. Calcium‐free superfusate solution and 10^−3^ M SNP produced vasodilations that were not significantly different (SNP [*n* = 9]: 23.2 ± 4.6 μm, EGTA [*n* = 9]: 23.4 ± 4.6 μm) with no significant order effect regarding agonist application. Therefore, 10^−3^ M SNP was considered to produce maximal vasodilation in females and was used throughout all subsequent experiments.

#### 2.4.6 In vitro retractor contraction experiments

Briefly, the left and right retractor muscles were isolated from euthanized male and female hamsters and placed in Krebs–Henseleit solution (NaCl [118 mM], KCl [4.7 mM], KH_2_PO_4_ [1.2 mM], MgSO_4_·7H_2_O [1.2 mM], NaHCO_3_ [27.26 mM], glucose [11.1 mM], CaCl_2_·2H_2_O [2.3 mM], insulin [10 U/L] [Humulin R, Eli Lilly, Toronto, ON, Canada], and tubocurarine [0.3 mg/L]) for further dissection at room temperature. Insulin was added to the solution to facilitate muscle glucose uptake and curare was added to ensure muscle stimulation via the electrode itself and not through motor nerves. Suture silk ties (4.0 gauge) were secured to the muscle's origin and near its insertion into the cheek pouch. Two more suture silk ties were placed in the central region, and the muscle was cut in half to elicit 2 strips of muscle from each retractor; therefore 4 strips total per 2 retractor muscles. Muscle strips were suspended vertically in a stimulating apparatus whereby the distal tie was attached to a fixed hook and the proximal tie was attached to a force transducer (Grass Instruments, Quincy, MA, USA). Stainless steel stimulating electrodes connected to a stimulator (Grass Instruments, Quincy, MA, USA) were placed on either side of the muscle strips, but not touching the muscle, to elicit muscle contraction via field stimulation. The muscle strips were immersed in a glass organ bath chamber filled with Krebs–Henseleit (pH 7.35–7.45, at 27°C), and continuously aerated with 95% O_2_–5% CO_2_. In a sub‐set of in vitro experiments, maximal stimulus frequency was determined to be 70 Hz. Muscles were set to optimal length using 70 Hz and equilibrated for 45–60 min prior to each experimental protocol.

Retractor muscles were stimulated to contract isometrically using a supramaximal voltage (100 V) with a 250 ms train duration during (i) force‐frequency test, (ii) twitch contraction with data collected at a high sampling rate (500 Hz), and (iii) during 2 min contraction protocols (protocol A or B) as indicated in the above in situ muscle contraction experiments. The force‐frequency relationship was determined by stimulating the muscle at randomized stimulus frequencies of 1, 5, 10–120 (in 10 Hz increments) at 1 cpm for each muscle. Female and male retractor muscles were then randomized to contraction protocol A: stimulus frequencies at 4, 20, and 70 Hz at a constant contraction frequency of 15 cpm or contraction protocol B: contraction frequencies at 6, 15, and 60 cpm at a constant stimulus frequency of 20 Hz. Contraction conditions within each contraction protocol were completed in a randomized order and there were 5 min of recovery between contraction bouts. Care was taken to ensure that the two muscle strips from the same retractor muscle underwent different contraction protocols. Muscle wet weights and optimal lengths were obtained following the cessation of the experiment.

### 2.5 Data analysis and statistics

#### 2.5.1 In situ experiments

Baseline diameters were recorded constantly for 1 min during collection periods and were defined as the diameter of the arteriole after 1 min. For vascular reactivity experiments in response to muscle contractions and pharmacological agonists, arteriolar diameters were constantly recorded for 1 min prior to muscle contractions or the addition of the agonist (baseline), during the 2 min contraction period or agonist exposure period, and for 2 min during the cessation of muscle contractions or agonist wash‐out period with no agonist exposure. In these experiments, baseline diameter was defined as the diameter of the arteriole after 1 min just prior to muscle contractions or agonist exposure. Arteriolar diameters were measured every 10 s during all in situ experiments and still images were captured every 10 ± 1 s using FrameShot software (EoF Productions, Sacramento, CA, USA). Arteriolar diameters were measured using ImageJ (NIH, Bethesda, MD, USA).

Only one arteriole per arteriolar level was observed when assessing baseline and maximum diameters across the arteriole network, with *n* indicating the number of arterioles observed. For vascular reactivity experiments, only one 3A arteriole was stimulated, either physiologically or pharmacologically, per retractor preparation. In each of these experiments, *n* indicates the number of 3A arterioles observed.

#### 2.5.2 In vitro experiments

Isometric force data were collected and analyzed using the MP100WSW data acquisition system and Acqknowledge III software (Biopac Systems Inc., Goleta, CA, USA) on an IBM computer. For the force‐frequency test, retractor force generation in males and females were normalized to wet muscle weight (mg) and to a percentage of maximum force generated. Force generated during the 2 min contraction protocols was normalized to a percentage of force generated during the initial isometric contraction. Given that two retractor muscles were excised and bisected from each hamster yielding 4 strips, each strip would have undergone the force‐frequency test and 2 strips would have undergone each contraction protocol (A or B) at random. For the force‐frequency curve and contraction protocols, only 1 muscle strip from each animal (chosen at random) was included in the data set for statistical analysis.

#### 2.5.3 Statistics

All data are reported as mean ± standard deviation (SD). Graphical and statistical analyses were completed using GraphPad (Prism 9, GraphPad Software Inc). For the in situ experiments, an unpaired *t*‐test was used to compare group means when only 2 means were reported (i.e., baseline diameters, maximum diameters, distance between local stimulation site and upstream observation site, SNP vs. calcium‐free control experiments and rate data). Linear regression was used to measure the rate of contraction over the first 60 s of agonist application or muscle contraction and the half‐recovery rate following the cessation of stimulation. A two‐way ANOVA was used to compare rate and magnitude data (vasodilation rate, half recovery rate and maximum change in diameter to a stimulus). A two‐way repeated measures ANOVA was used to compare group means when the data were reported over time (i.e., vascular reactivity data [vasodilation and recovery in response to muscle contractions and pharmacological agonists]). For the in vitro control experiments, sex differences in force production were analyzed using a two‐way ANOVA (force‐frequency data) and a two‐way repeated measures ANOVA when group means were reported over time during the 2 min isometric contraction protocols. A protected least squared difference test was used for post hoc analysis when the ANOVA identified a significant main effect. Differences were considered significant when *p* < 0.05.

## 3 RESULTS

### 3.1 In situ vascular reactivity experiments

#### 3.1.1 Resting baseline diameters and maximum diameters across the arteriolar network

The arteriolar microvascular network did not differ structurally between males and females. In both sexes, 3 arteriolar branch orders were identified between the paired 1A arteriole and the capillary unit. Baseline and maximal diameters across 2A, 3A, and 4A arterioles were not significantly different between females and males (Table 1).

**TABLE 1:** Average baseline and maximum diameters of 2A, 3A, and 4A arterioles did not differ between male and female retractor muscles.

	2A		3A		4A	
	Female	Male	Female	Male	Female	Male
Baseline diameter (μm)	23.5 ± 3.0	24.0 ± 2.7	13.2 ± 2.9	13.0 ± 2.8	7.6 ± 0.8	7.6 ± 1.9
Maximum diameter (μm)	38.7 ± 4.6	41.7 ± 5.1	22.5 ± 5.0	21.9 ± 3.7	13.2 ± 1.7	12.3 ± 1.8

*Note*: *n* for each arteriolar level: 2A arteriole (male *n* = 7, female *n* = 7), 3A arteriole (male *n* = 9, female *n* = 8), and 4A arteriole (male *n* = 6, female *n* = 6).

#### 3.1.2 Arteriolar reactivity in response to muscle contractions

Baseline and maximal diameters of 3A arterioles did not significantly differ in muscle contraction experimental protocols between females and males (Table 2).

**TABLE 2:** Average baseline and maximal diameters did not differ significantly between males and females during the muscle contraction experimental protocols.

	Baseline diameter (μm)		Maximal diameter (μm)	
	Female	Male	Female	Male
Protocol A: altered stimulus frequency (at 15 cpm)				
4 Hz	12.3 ± 2.5	14.2 ± 1.4	24.4 ± 5.9	22.8 ± 5.2
20 Hz	12.0 ± 1.7	13.3 ± 1.6	23.8 ± 5.0	23.2 ± 4.7
70 Hz	12.5 ± 2.4	13.3 ± 2.5	23.1 ± 3.6	23.2 ± 4.8
Protocol B: altered contraction frequency (at 20 Hz)				
6 CPM	12.4 ± 1.5	13.8 ± 3.4	23.4 ± 3.8	22.7 ± 4.4
15 CPM	12.1 ± 1.2	13.4 ± 3.0	24.5 ± 2.1	23.5 ± 4.3
60 CPM	12.1 ± 1.7	13.9 ± 4.3	23.8 ± 3.4	22.7 ± 5.2

*Note*: *n* for each contraction condition within each protocol: Protocol A (at 15 cpm): 4 Hz (males *n* = 6; females *n* = 6), 20 Hz (males *n* = 9; females *n* = 9), and 70 Hz (males *n* = 7, females *n* = 7). Protocol B (at 20 Hz): 6 cpm (males *n* = 10; females *n* = 7), 15 cpm (males *n* = 10, females *n* = 7), and 60 cpm (males *n* = 10, females *n* = 9).

Abbreviation: CPM, contractions per minute.

**FIGURE 2:**
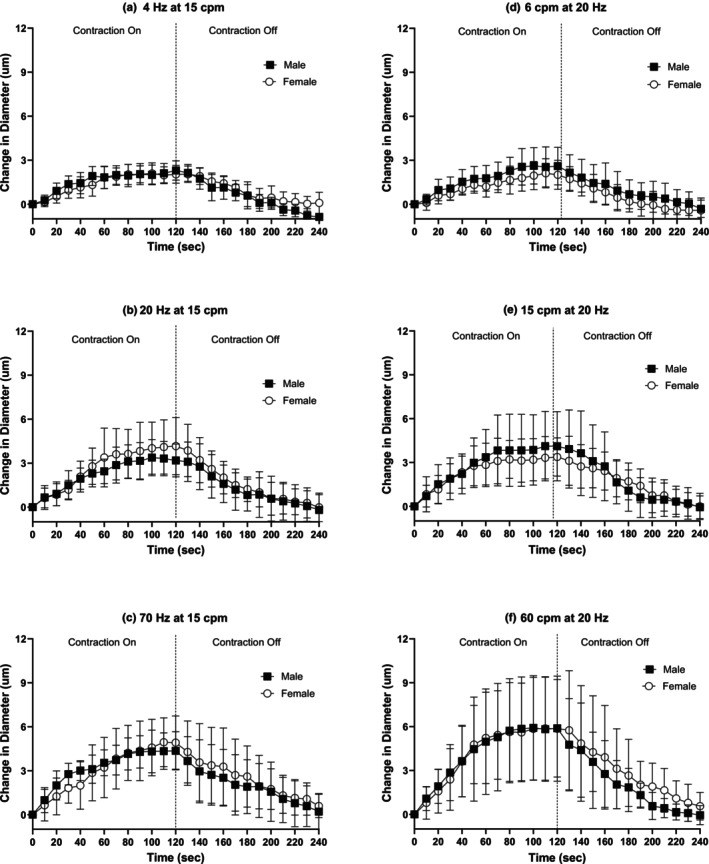
No sex differences were observed in 3A arteriolar vasodilation in response to muscle contractions. 3A arteriolar reactivity in response to muscle contraction during twitch and tetanic contractions for 2 min (0–120 s) and 2 min (120–240 s) of recovery following the cessation of contractions in males (black squares) and females (open circles). Muscle contraction protocols: protocol A at 15 cpm at (a) 4 Hz, (b) 20 Hz and (c) 70 Hz, and protocol B at 20 Hz at (d) 6 cpm, (e) 15 cpm and (f) 60 cpm. Baseline diameters, maximum diameters, and *n's* for each protocol can be found in Table 2.

Muscle contraction protocols induced vasodilation locally at the 3A arteriole under all stimulation conditions over the course of the 2 min contraction bout in females and males (Figure 2). Following cessation of muscle contractions, arteriolar diameter returned to baseline under all contraction conditions by 2 min in both females and males. No significant sex differences were observed in the 3A arteriolar vasodilatory response between males and females during any muscle contraction protocol or during recovery (Figure 2a–f). No sex differences were found in the rate of vasodilation and half‐recovery rate under any stimulation condition (Table 3).

**TABLE 3:** Average vasodilation rate during the first 60 s of contractions or pharmacological agonist application and the average half recovery rate following the cessation of muscle contractions or pharmacological agonist.

Experiment condition	Dilation rate (μm/s)		Half recovery rate (μm/s)	
	Female	Male	Female	Male
**Drug application**				
10^−5^ M NO	0.11 ± 0.05	0.11 ± 0.05	−0.06 ± 0.07	−0.06 ± 0.06
10^−6^ M ADO	0.08 ± 0.03	0.06 ± 0.04	−0.05 ± 0.06	−0.04 ± 0.08
20 mM K^+^	0.02 ± 0.01	0.04 ± 0.04	−0.02 ± 0.04	−0.02 ± 0.07
10^−6^ M ACh–local	0.22 ± 0.14	0.18 ± 0.11	−0.09 ± 0.17	−0.10 ± 0.11
10^−6^ M ACh–upstream	0.13 ± 0.12	0.13 ± 0.13	−0.04 ± 0.07	−0.03 ± 0.03
**Muscle contraction**				
15CPM 4 Hz	0.03 ± 0.01	0.03 ± 0.01	−0.02 ± 0.02	−0.03 ± 0.02
15CPM 20 Hz	0.06 ± 0.02	0.04 ± 0.01	−0.06 ± 0.05	−0.04 ± 0.04
15CPM 70 Hz	0.05 ± 0.03	0.06 ± 0.02	−0.04 ± 0.05	−0.04 ± 0.04
20 Hz 6CPM	0.02 ± 0.01	0.03 ± 0.01	−0.03 ± 0.04	−0.03 ± 0.04
20 Hz 15CPM	0.05 ± 0.02	0.05 ± 0.02	−0.03 ± 0.03	−0.05 ± 0.06
20 Hz 60CPM	0.09 ± 0.04	0.08 ± 0.04	−0.05 ± 0.1	−0.07 ± 0.09

### 3.2 Arteriolar reactivity in response to pharmacological agonists

- Arteriolar reactivity in response to NO, ADO, and K^+^

**FIGURE 3:**
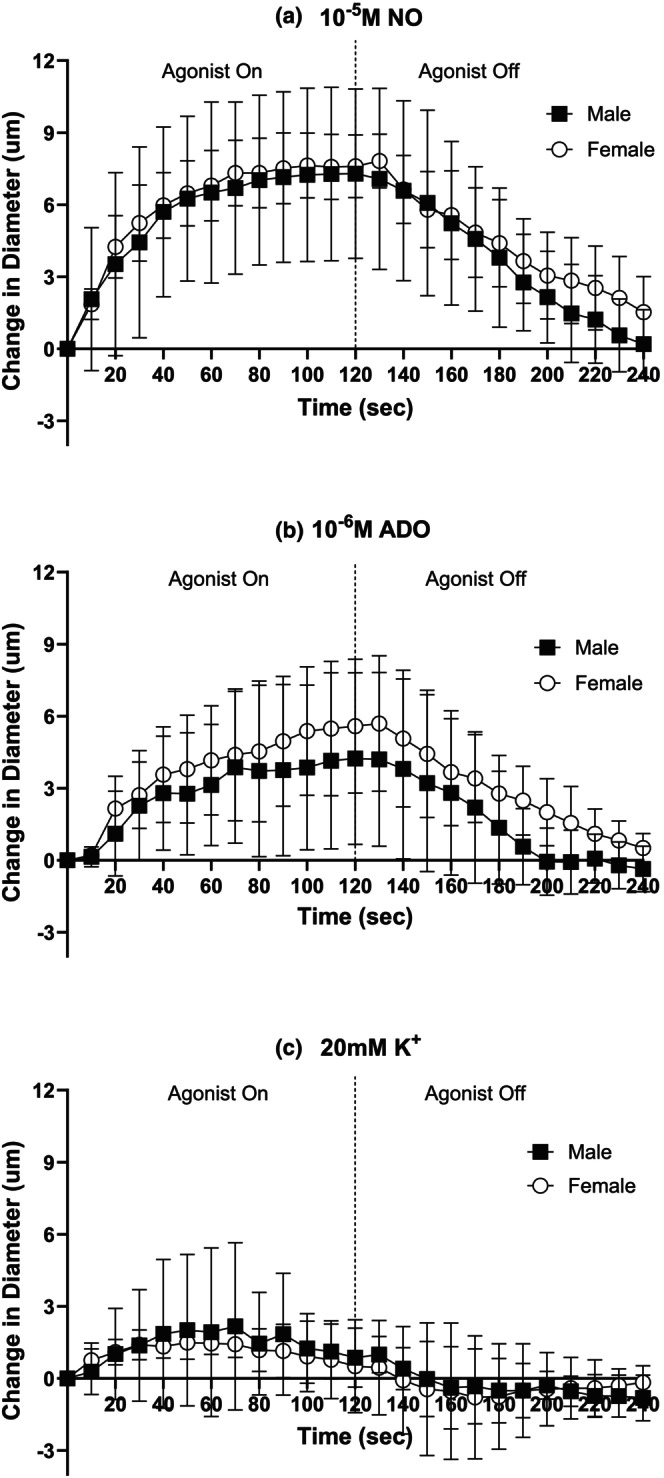
3A arteriolar reactivity to NO, ADO, and K^+^ did not differ between males and females. 3A arteriolar response to micropipette application with (a) 10^−5^ M NO, (b) 10^−6^ M ADO, and (c) 20 mM K^+^ for 2 min (0–120 s) and 2 min (120–240 s) of recovery following the cessation of agonist application in males (black squares) and females (open circles). Baseline diameters, maximum diameters, and *n's* for each protocol can be found in Table 4.

Baseline and maximal diameters did not significantly differ over the duration of the micropipette experimental protocols in females and males (Table 4). In females and males, each agonist (10^−5^ M NO, 10^−6^ M ADO, and 20 mM K^+^) elicited local vasodilation at the 3A arteriole that returned to baseline after 2 min of wash‐out (Figure 3a–c). No significant sex differences were observed in 3A arteriolar reactivity to NO (Figure 3a), ADO (Figure 3b), or K^+^ (Figure 3c) during exposure to the agonist or during recovery. No sex differences were found in the rate of vasodilation and half‐recovery rate in response to NO, ADO, or K^+^ (Table 3).

ii Local and conducted responses induced by ACh

**FIGURE 4:**
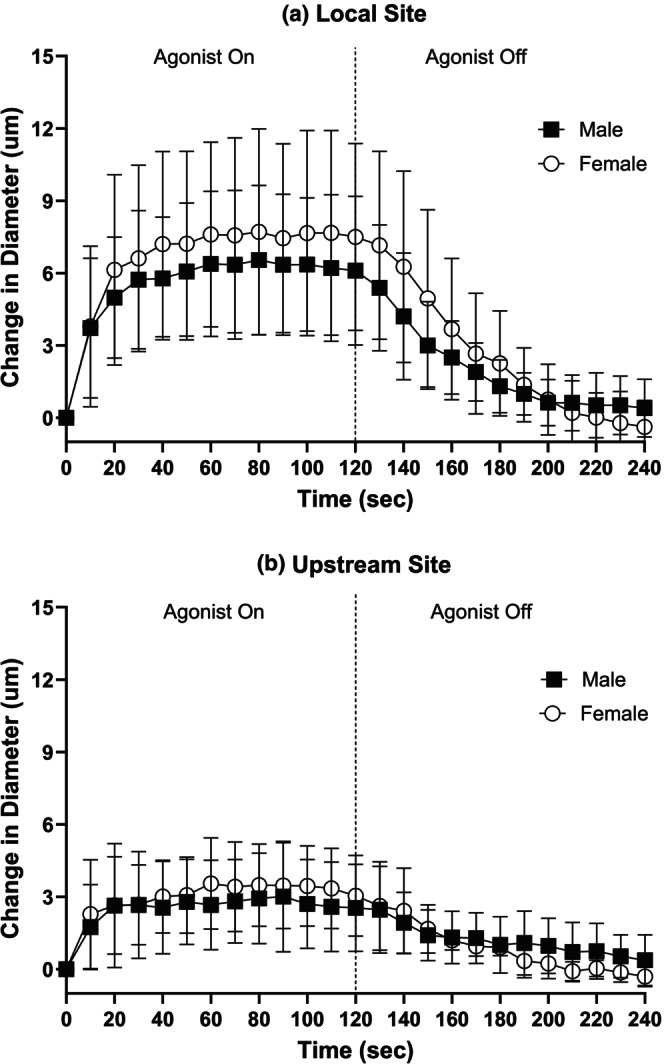
3A arteriolar reactivity to ACh at the local and upstream observation site was not different between males and females. 3A arteriolar reactivity to 2 min exposure (0–120 s) to 10^−6^ M ACh 2 min (120–240 s) of recovery following the cessation of agonist application in males (black squares) and females (white circles) at the (a) local stimulation site and (b) upstream observation site. Males (local site *n* = 9 and upstream site *n* = 8) and females (local site *n* = 8 and upstream site *n* = 6).

Baseline and maximal diameters at the local stimulation site and upstream observation site on the 3A arteriole did not significantly differ between males and females. At the local 3A stimulation site, the average resting baseline diameter in males was 13.0 ± 2.8 μm (*n* = 9) versus 13.2 ± 2.9 μm in females (*n* = 8), and the average maximal diameter was 21.9 ± 3.7 μm in males versus 22.5 ± 5.0 μm in females. At the upstream 3A observation site, the average resting baseline diameter in males was 11.9 ± 3.1 μm (*n* = 8) compared to 11.0 ± 1.7 μm in females (*n* = 6), and the average maximal diameter was 22.2 ± 4.6 μm in males versus 21.7 ± 6.3 μm in females. The average distance between the local stimulation site and upstream observation site did not significantly differ between males and females (male distance: 761.8 ± 262.3 μm vs. female distance: 836.2 ± 254.0 μm). In females and males, 2 min of 10^−6^ M ACh application resulted in a local vasodilation and an upstream conducted response that fully recovered back to baseline after 2 min of wash‐out (Figure 4). No significant differences were observed between females and males in the magnitude of the local vasodilation (Figure 4a) or upstream conducted response (Figure 4b) during 2 min of ACh exposure. No sex differences were found in the rate of vasodilation and half‐recovery rate in response to ACh at the local and upstream site (Table 3).

**TABLE 4:** Average baseline and maximum diameters at the local 3A arteriolar stimulation site did not significantly differ in males and females for the experiments involving micropipette application of NO, ADO, or K^+^.

	NO		ADO		K^+^	
	Female	Male	Female	Male	Female	Male
Baseline diameter (μm)	11.9 ± 1.0	12.5 ± 2.0	11.4 ± 0.8	12.4 ± 2.2	11.5 ± 1.9	12.9 ± 2.2
Maximum diameter (μm)	23.5 ± 6.1	22.2 ± 2.8	23.4 ± 5.5	22.7 ± 2.7	23.1 ± 5.8	22.8 ± 2.6

*Note*: *n* for each pharmacological agonist: 10^−5^M NO (male *n* = 7, female *n* = 6), 10^−6^ M ADO (male *n* = 7, female *n* = 7), and 20 mM K^+^ (male *n* = 8, female *n* = 7).

### 3.3 In vitro retractor contraction experiments

**FIGURE 5:**
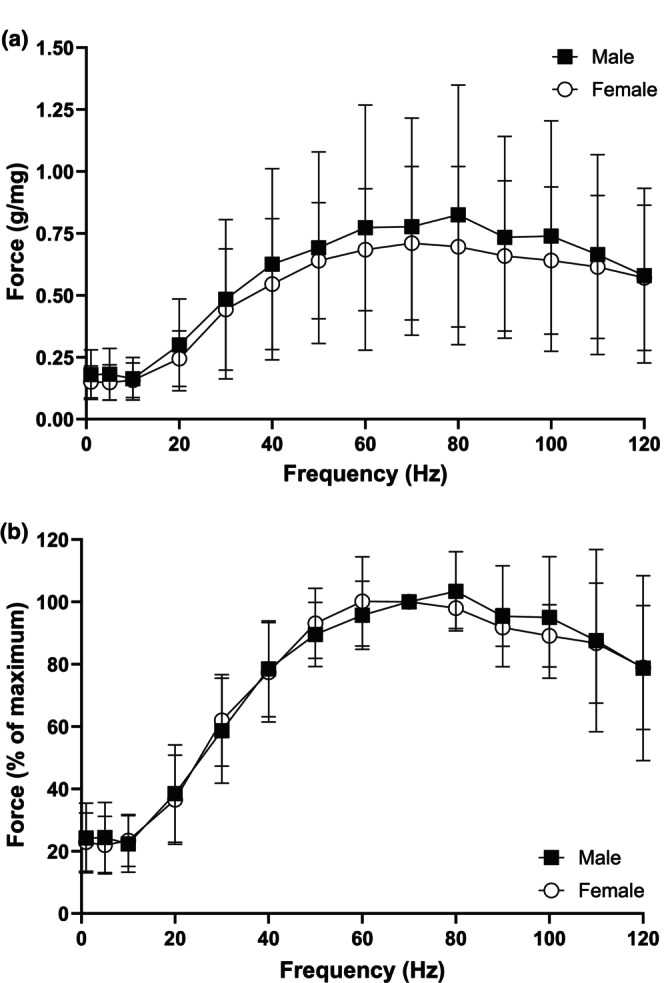
Sex differences were not apparent in force production during twitch or tetanic contractions in the retractor muscle in vitro. Force generated during the force‐frequency curve at 1, 5, 10–120 Hz stimulus frequencies in males (black squares, *n* = 5) and females (white circles, *n* = 5). Force was normalized to wet muscle weight (a) or to a percentage of maximum force (b).

**FIGURE 6:**
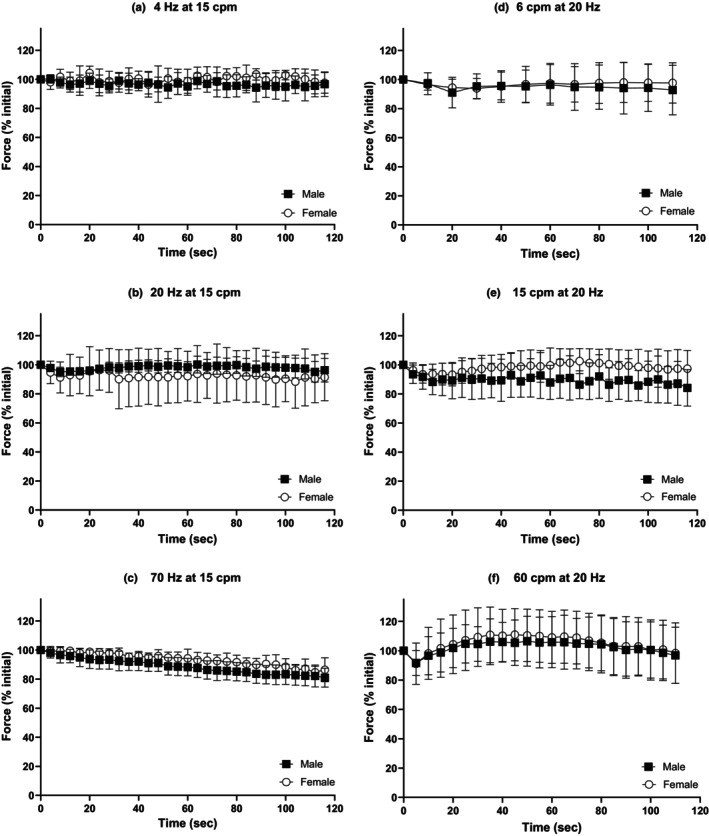
Force production during 2 min of contractions did not differ between males and females under any contraction protocol in vitro. Male (black squares) and female (open circles) retractor force production during 2 min isometric contraction protocols in vitro. Contraction protocols used a 250 ms train duration. Protocol a: at 15 cpm at (a) 4 Hz, (b) 20 Hz, and (c) 70 Hz. Protocol b: at 20 Hz at (d) 6 cpm, (e) 15 cpm and (f) 60 cpm. *n's* for each contraction condition within each contraction protocol were as follows: Males *n =* 5 for all conditions, females *n* = 5 for all conditions except at 15 cpm at 4 Hz *n* = 4.

Sex differences in force generation were not present at any stimulus frequency during analysis of the force‐frequency curve when normalized to wet muscle mass (specific force) (Figure 5a) or when normalized to a percentage of maximum force (Figure 5b). Twitch characteristics of the retractor muscle did not differ between males and females. There were no significant differences in specific twitch force, time to peak force and half‐relaxation time (Table 5). Lastly, force generation over the 2 min contraction periods did not differ between males and females during muscle contraction protocols (Figure 6a–f).

**TABLE 5:** Twitch characteristics of retractor muscle in vitro did not differ in females and males.

	Female (*n* = 5)	Male (*n* = 5)
Peak force (g)	2.3 ± 1.0	2.6 ± 1.4
Specific force (g/mg)	0.13 ± 0.07	0.14 ± 0.08
TTP (ms)	54.8 ± 3.3	57.2 ± 6.1
½RT (ms)	26.8 ± 1.1	29.2 ± 3.6

*Note*: TTP (time to peak twitch force) and ½RT (time to half relaxation).

## 4 DISCUSSION

The main objective of this study was to determine if sexual dimorphisms were present in arteriolar reactivity with regards to processes important to the blood flow response during muscle contractions. To investigate this aim, we explored if sexual dimorphisms were present in microvascular reactivity initiated by a physiological stimulus, muscle contraction, and pharmacological agents relevant to muscle contraction (NO, ADO, and K^+^) and those that induce conducted responses (ACh). The main findings from this study were that vasodilatory arteriolar reactivity in response to muscle contractions over a wide range of twitch and tetanic contractile conditions did not differ between females and males. No differences were found in the magnitude of the vasodilations in response to muscle contractions, the rate of vasodilation or the rate of recovery of vasodilation following the cessation of contractions. Similarly, no sexual dimorphisms were observed in the magnitude and rates of vasodilation and rates of recovery with direct stimulation with NO, ADO, and K^+^. Finally, no differences in the local or conducted responses to ACh were observed. These data imply that, at the local level, the microvascular control mechanisms in arterioles that govern the vascular response to muscle contractions do not appear to be sexually dimorphic. Further, if there are sexually dimorphic components, they are complementary and matched between males and females such that the integrated response results in vasodilatory responses that do not differ.

### 4.1 Baseline and maximal diameters in the arteriolar network

In the current study, resting baseline and maximal diameters across 2A, 3A, and 4A arterioles did not differ between females and males (Table 1). Similar resting baseline diameters and maximal diameters have been documented between females and males in larger arteries in the brain (Geary et al., 2000; Mayhan et al., 2002; Sun & Mayhan, 2005) and heart (Heaps & Bowles, 2002; Wong et al., 2014), in smaller first order arterioles in the diaphragm (Horn, Morrison, et al., 2025; Horn, White, et al., 2025) and in second order arterioles within skeletal muscle (Huang et al., 1997) in rodent models in vivo and in vitro. In human models, sexual dimorphisms in resting arterial tone have been identified whereby females have a smaller diameter than males (Divia Paul et al., 2018; Green et al., 2025; Miller et al., 2019; Sullivan et al., 2015; Wahood et al., 2022) that persists, at least in the coronary arteries, when sexual dimorphisms were normalized to anthropometric differences between males and females (Dickerson et al., 2010; Hiteshi et al., 2014). Interestingly the differences in vessel size only persisted in proximal segments and not distal segments of the vasculature (Dickerson et al., 2010). Therefore, sexual dimorphisms in baseline diameter may be greater at the macrovascular level than at the microvascular level. Importantly, experimentally, similarities in the anthropometrics of male and female Golden Syrian Hamsters and no differences in the microvascular arteriolar baseline and maximum diameter between females and males in the current studies indicated that no adjustments or normalizations in the present data were required for male/female comparisons and, further, indicate arterioles have similar capacities to dilate when stimulated pharmacologically or physiologically.

Arterioles that are not actively being stimulated to change their diameter are considered to be at “rest” and exhibit a baseline diameter that is partially constricted, referred to as having baseline tone. Tone is dictated by the net sum of the vasodilatory and vasoconstrictor inputs that act on the vascular smooth muscle cells (Kerage et al., 2014; Korthuis, 2011; Nyberg et al., 2015). The multiple factors involved in tone development are derived from central (neural or hormonal influences) and local (autocrine, paracrine, the myogenic response and mechanical factors, that is, wall shear stress) sources (Bassenge & Busse, 1988; Delp & Laughlin, 1998; Halvorson et al., 2023; Jackson, 2000; Korthuis, 2011). No differences in the levels of basal tone in 2A, 3A, and 4A arterioles between sexes in our study indicate that either: (1) the sum total of the inputs acting on the vascular smooth muscle cells are not different between females and males or (2) that the complement of inputs are different and have varying degrees of influence whereby the net outcome results in diameters that are not different between the sexes. The latter is a possibility given the potential for sexually dimorphic differences in neural and hormonal influences on blood vessels. For example, it has been demonstrated that women have been shown to have lower resting muscle sympathetic nerve activity (MSNA) than men where MSNA is correlated to peripheral vasoconstriction in men but not women (Hart et al., 2009, 2011) and that beta‐adrenergic mediated vasodilation is enhanced in women compared to men (Hart et al., 2011; Kneale et al., 2000). Both of these differences suggest a reduced sympathetic mediated vasoconstriction in females at rest.

Further, sexual dimorphisms have been observed in the vascular response to circulating hormones such as norepinephrine and epinephrine (for review see (Orshal & Khalil, 2004; Pabbidi et al., 2018)) (Crews & Khalil, 1999; Kneale et al., 2000; Majmudar et al., 2000; Stallone et al., 1991), vasopressin (Stallone et al., 1991), endothelin (Gillis et al., 2016; Kellogg Jr. et al., 2001; MacIntyre et al., 2012), and angiotensin II (De Silva et al., 2009; Girouard et al., 2008) and females have a greater estradiol and progesterone vasodilatory influence on vascular endothelial cell function than in males (for review see (Pabbidi et al., 2018; Robert, 2023)). Finally, in the rat tail artery, wall shear stress plays a greater role in vasodilation in females than males (Pak et al., 2002). Therefore, if females are less sensitive to certain vasoconstrictor inputs and have greater vasodilatory influences, then we might expect females to have a greater resting diameter than males, but we did not observe this. Therefore, either females have different influences on basal tone than males but the integrated sum total of the differences results in a baseline tone that does not differ from males, or there are no differences in the influences on baseline diameter between males and females at the microvascular level. Clearly, more work will be required to determine if the contributors to basal tone are, in fact, not different between males and females at the microvascular level.

### 4.2 Vascular response to muscle contractions

The vascular response to muscle contraction is a complex integration of signaling requiring multiple vasodilatory influences and processes, especially over the 2‐min contraction period used in the current experiments. With the muscle fiber bundle stimulation model used here we expect there to be little sympathetic nervous system vasoconstriction involvement as there is no change in mean arterial pressure when stimulating so few muscle fibers (Dua et al., 2009). We do expect primarily local processes to contribute to the arteriolar vasodilation including metabolic vasodilator production and release resulting from contracting skeletal muscle, along with other diffusible factors from different sources (i.e., endothelial cells and red blood cells), flow‐mediated vasodilation, functional sympatholysis, the myogenic response, and conducted responses from other parts of the vasculature. We found no differences in the magnitude of vasodilation and the rates of vasodilation and recovery from vasodilation under any twitch or tetanic contractile condition studied. This was surprising given the potential for differences in the processes that contribute to vasodilation in response to muscle contractions where sex differences have been identified, including: (1) the potential sex differences in muscle contractile properties/processes that yield the vasodilators, (2) the potential sex differences in the vascular response to the same vasodilators, (3) the potential sex differences in conducted responses and other modifying vasodilatory processes involved in the vasodilatory response to muscle contraction.

1. Muscle contractile properties/processes

Force generation and ATP utilization in active skeletal muscle will drive metabolism of the muscle and the generation of metabolic vasodilators. If contractile force and/or metabolism differs between males and females then the resulting vasodilators may also be different. Sex differences in muscle‐specific force (force normalized to muscle weight) have been demonstrated in vitro (Bisschop et al., 1997; Lafoux et al., 2020) but not consistently (Hill et al., 2020; Khurram et al., 2018) and there are reports of sex differences in resting metabolic rate (Ansdell et al., 2019; Citherlet et al., 2024; Fellahi et al., 2014; Keller et al., 2023; Landers‐Ramos et al., 2024; Molbo et al., 2022; Rasica et al., 2022; Zaleski et al., 2023) and metabolic properties (Carter et al., 2001; Miotto et al., 2018; Tarnopolsky, 2008), therefore, there is the possibility that males and females produce different metabolic vasodilators. We, however, saw no evidence of these differences. We would have expected that if different vasodilators were released during contractions then the magnitude of vasodilation, the rate of vasodilation and the rate of recovery of vasodilation would be different between males and females, but we saw no evidence suggesting this over a wide range of contractile conditions.

To confirm that there were no differences in contractile force between females and males, we compared specific force and fatigue characteristics of the retractor muscle in vitro. We observed that male and female muscles had no significant differences in twitch contraction characteristics, specific force production over a range of tetanic stimulus frequencies, or change in force over time when stimulated with a range of contraction and stimulus frequencies. Male retractor muscle fiber type proportions have been reported as approximately 15% type I, 27% type IIA, 58% type IIX with negligible type IIB fibers (Mattson et al., 2002). While we did not perform fiber typing in this study of the retractor muscle, our twitch data indicate no significant differences in contractile characteristics between males and females suggesting no differences in fiber types (Table 5). The lack of differences in contractile characteristics and force generation between male and female retractor muscles supports that metabolism between male and female retractor muscles may not be different and therefore may be generating similar metabolic vasodilators. Future work focusing on muscle metabolism will be required to confirm these suppositions.

2 Arteriolar vascular response to vasodilators

We confirmed the lack of sex differences in local arteriolar vascular reactivity to a given vasodilator by directly exposing arterioles to sub‐maximal concentrations of vasodilators thought to be relevant to the active hyperaemic process (ADO, NO, and K^+^) (Joyner & Casey, 2015; Ross et al., 2013; Sarelius & Pohl, 2010). We found no differences in the magnitude of the vasodilation, the rate of vasodilation, and the rate of vascular recovery following cessation of vasodilator application for each vasodilator tested (Figure 3, Tables 3 and 4). It is possible that sexual dimorphic vascular responses are dependent on the size or vascular level of the vessel (Bell et al., 1995; Dias et al., 2014; Heaps & Bowles, 2002; Hester et al., 1993; Horn, White, et al., 2025; Huang et al., 1997; Kauser & Rubanyi, 1994; Kauser & Rubanyi, 1995; Mayhan et al., 2002; Prentice & Hourani, 2000; Ray & Marshall, 2006; Rose'Meyer & Hope, 1990; Sanchez et al., 1996; Sarabi et al., 1999; Sun & Mayhan, 2005). For example, in large arteries such as the aorta of rats, females have been shown to be more reactive to K^+^ at concentrations <10 mM (Dias et al., 2014), but males are more responsive than females at concentrations of 10–20 mM (Shechtman & Katovich, 1993) while there was a lack of sex differences observed in smaller, more distal blood vessels in response, in the rat tail artery (Shechtman & Katovich, 1993), retinal arterioles (MacIntyre et al., 2012) and first order arterioles in the medial costal diaphragm (Horn, Morrison, et al., 2025). Therefore, sexual dimorphisms in vascular reactivity to vasodilators may dissipate from the macro‐ to the microvasculature. This trend may not be true for all vasodilators as there appears to be a lack of sex differences in the response to NO donors across many levels of the vasculature from the aorta (Dias et al., 2014) to arteries (Bell et al., 1995; Dias et al., 2014; McCulloch & Randall, 1998; Skarsgard et al., 1997) to arterioles (Horn, White, et al., 2025), although not invariably (Mayhan et al., 2002). Our lack of sex differences to ADO, NO, and K^+^, using 3A arterioles, two arteriolar levels upstream from the capillaries in the retractor muscle, supports the idea that sex differences in vascular response to agonists may be less apparent in the distal arteriolar microvasculature. Indeed, the heterogeneous nature of endothelial cells (Huxley et al., 2018), especially along the length of a vessel (McCarron et al., 2019; Mrazkova et al., 1986) could facilitate this behavior.

Sex differences at the microvascular level may lie, not in the absolute response to the agonist, but in the intracellular, cell signaling pathways producing the response. Wang et al. showed, using cultured microvascular endothelial cells from skeletal muscle, that the change in permeability induced by ADO was not different between the sexes, but the intracellular phosphodiesterase signaling pathway was different (Wang et al., 2010), indicating that females and males had different intracellular pathways to get to the same endpoint. Critical future work will need to focus on the cell‐signaling responses to agonists and muscle contraction at the arteriolar level to determine if sex differences lie intracellularly.

3 Conducted responses and other modifying processes involved in the vasodilatory response to muscle contractions

Conducted responses are an important mechanism by which blood vessels communicate longitudinally, transmitting a change in membrane potential from cell to cell through gap junctions, along the length of one vessel and into different branch orders of the microvasculature. This spread of vasodilation throughout the microvascular network is an important component required to coordinate the vascular response to muscle contraction (Berg et al., 1997). ACh was initially used to study conducted responses and is considered the gold standard in initiating and studying this phenomenon (for review see (Bagher & Segal, 2011)). We used ACh to investigate sex differences in the conducted responses and found no sexual dimorphisms in either the local or conducted vascular response to ACh (the magnitude of dilation, the rate of vasodilation and the rate of recovery of vasodilation after the cessation of ACh application). These data indicate vascular reactivity to endothelial cell muscarinic cholinergic stimulation did not differ between females and males, and that the capacity to conduct signals longitudinally along the vascular network was not sexually dimorphic.

As with K^+^, sex differences in vascular reactivity to muscarinic agonists (i.e., ACh and methacholine) may also dissipate from the macro‐to the microvasculature. Sex differences in response to ACh have been documented in the aorta (Dias et al., 2014; Kauser & Rubanyi, 1994; Kauser & Rubanyi, 1995; Sanchez et al., 1996), large arteries in the brain (Mayhan et al., 2002), mesentery (White et al., 2000) and brachial artery (Sarabi et al. 1999) while Sun and Mayhan show no differences in the more distal pial arteries (Sun & Mayhan, 2005), and Horn et al. (Horn, White, et al., 2025) show no differences in smaller, 1A arterioles in the diaphragm. Our lack of sexual dimorphisms with ACh at the 3A arteriolar level would support this trend and we add that the conducted response as a result of ACh stimulation of the arterioles does not differ between the sexes either. Interestingly, as with ADO, the intracellular cell‐signaling response to ACh may differ between sexes as vasodilation to ACh in the larger rat tail artery has been shown to be more dependent on EDHF in females compared to males (Pak et al., 2002), but whether these sex differences exist at the microvascular level will require further investigation.

### 4.3 Experimental considerations

#### 4.3.1 Retractor in vitro force production

Preparation of the retractor muscle for the in vitro protocols required the origin and insertion of the muscle to be severed and the ends of the muscle strips to be tied. This process results in damage to skeletal muscle fibers at the end of each strip. In calculating the force generated per milligram of muscle, these damaged fibers would contribute to the weight of the muscle but would not have contributed to the force of the muscle; therefore, the reported specific force generated by the retractor will be an underestimation of the actual force generated by the intact muscle fibers. Despite damaged muscle fibers, our retractor strips, as with other muscle strip preparations (Dua et al., 2009), generated consistent force with low variability in both females and males during the force‐frequency test and during the 2 min contraction protocols, thus proving to be a functionally viable in vitro preparation to discern sex differences in force production and muscle performance.

Hamster retractor muscle is a thin muscle, ranging from <150 μm at the edges to <600 μm at the centre in animals weighing ~80–100 g (Sullivan & Pittman, 1982). Segal and Faulkner (Segal & Faulkner, 1985) calculated the critical radius for oxygen diffusion in vitro to be ~0.9 mm at ~27°C in cylindrical muscles. As our animals were larger (90–143 g for females and males), retractor thickness may have exceeded 600 μm, although we did not measure this directly in our study. Thus, we cannot rule out mild hypoxia in deeper fibers during contractions in vitro. However, we minimized the potential of an anoxic core by maintaining preparations at 27°C and superfusing with 95% O₂ and glucose/insulin‐enriched buffer to support oxygen and energy delivery during contractions. Any hypoxic effects will have been comparable across sexes, as male and female animals did not differ in body weights in our study. Therefore, hypoxic effects would not affect our ability to compare male and female contractile performance.

#### 4.3.2 Force production, vascular reactivity, and the estrous cycle

The main objective of this study was not focused on hormonal influences on the microvasculature; therefore, the estrous cycle in female hamsters was not monitored (Shansky, 2019). However, we did monitor the variability between our female data in comparison to the male data throughout the in vitro and in situ experiments to determine if the estrous cycle may be influencing our results. For example, if there was larger variability within the female data sets compared to male data sets, then a biological factor may be influencing the female data that was not influencing the males, which would point us in the direction that perhaps the estrous cycle was impacting our results. However, the similarity in the variability in both the in vitro and in situ studies between males and females indicates that hormonal fluctuations during the estrous cycle in females were not affecting retractor force production or arteriolar reactivity. Therefore, our data provide no evidence to suggest that the estrous cycle was impacting our results in females or final conclusions from this study.

## 5 CONCLUSIONS

The main objective of this study was to investigate sexual dimorphisms in microvascular reactivity in response to muscle contractions and processes involved in microvascular blood flow control in skeletal muscle. We demonstrated that arteriolar reactivity to muscle contractions and to vasodilators known to be involved in muscle contraction (NO, ADO, and K^+^) were not significantly different between females and males. We show that despite using a wide range of contraction parameters, there were no differences in the arteriolar vasodilatory characteristics between males and females in the integrated response to muscle contractions. While there may be sex differences in active hyperaemic processes and intracellular signaling responses between males and females that were not tested here, these differences had to have balanced out to result in an integrated arteriolar response between the sexes that showed no differences. Future work will need to determine if there are in fact sexual dimorphisms in intracellular signaling and other mechanisms involved in the integrated blood flow response to muscle contractions. If there are in fact no sex differences in intracellular signaling processes, then it is possible that the microvasculature is less affected by sexual dimorphisms than the macrovasculature.

## FUNDING INFORMATION

NSERC Canada RGPIN‐2019‐05146.

## ETHICS STATEMENT

All procedures were approved by the Animal Care Committee at the University of Guelph and were performed in accordance with the Canadian Council of Animal Care guidelines.

## Data Availability

Data will be made available upon reasonable request.
